# Tenofovir, emtricitabine, lamivudine and dolutegravir concentrations in plasma and urine following drug intake cessation in a randomized controlled directly observed pharmacokinetic trial to aid point-of-care testing

**DOI:** 10.1093/jac/dkae147

**Published:** 2024-05-17

**Authors:** Laura J Else, Laura Dickinson, Stacey Edick, Ashley Zyhowski, Ken Ho, Leslie Meyn, Sujan Dilly-Penchala, Beth Thompson, Victoria Shaw, Saye Khoo, Rhonda M Brand

**Affiliations:** Department of Molecular and Clinical Pharmacology, University of Liverpool, Liverpool, UK; Department of Molecular and Clinical Pharmacology, University of Liverpool, Liverpool, UK; Division of Infectious Diseases, University of Pittsburgh School of Medicine, Pittsburgh, PA, USA; Magee-Womens Research Institute, Pittsburgh, PA, USA; Division of Infectious Diseases, University of Pittsburgh School of Medicine, Pittsburgh, PA, USA; Division of Infectious Diseases, University of Pittsburgh School of Medicine, Pittsburgh, PA, USA; Department of Molecular and Clinical Pharmacology, University of Liverpool, Liverpool, UK; Department of Molecular and Clinical Pharmacology, University of Liverpool, Liverpool, UK; Department of Molecular and Clinical Pharmacology, University of Liverpool, Liverpool, UK; Department of Molecular and Clinical Pharmacology, University of Liverpool, Liverpool, UK; Division of Infectious Diseases, University of Pittsburgh School of Medicine, Pittsburgh, PA, USA; Magee-Womens Research Institute, Pittsburgh, PA, USA

## Abstract

**Background:**

Poor adherence to ART and pre-exposure prophylaxis (PrEP) can impact patient and public health. Point-of-care testing (POCT) may aid monitoring and adherence interventions.

**Objectives:**

We report the pharmacokinetics of tenofovir [dosed as tenofovir disoproxil (TDF) and tenofovir alafenamide (TAF)], emtricitabine (FTC), lamivudine (3TC) and dolutegravir (DTG) in plasma and urine following drug cessation to evaluate adherence targets in urine for POCT.

**Methods:**

Subjects were randomized (1:1) to receive DTG/FTC/TAF or DTG/3TC/TDF for 15 days. Plasma and spot urine were collected on Day 15 (0–336 h post final dose). Drug concentrations were quantified using LC-MS, and non-linear mixed-effects models applied to determine drug disposition between matrices and relationship with relevant plasma [dolutegravir protein-adjusted 90% inhibitory concentration (PA-IC_90_ = 64 ng/mL) and minimum effective concentration (MEC = 324 ng/mL)] and urinary thresholds [tenofovir disoproxil fumarate 1500 ng/mL].

**Results:**

Of 30 individuals enrolled, 29 were included (72% female at birth, 90% Caucasian). Median (range) predicted time to plasma dolutegravir PA-IC_90_ and MEC were 83.5 (41.0–152) and 49.0 h (23.7–78.9), corresponding to geometric mean (90%) urine concentrations of 5.42 (4.37–6.46) and 27.4 ng/mL (22.1–32.7). Tenofovir in urine reached 1500 ng/mL by 101 h (58.6–205) with an equivalent plasma concentration of 6.20 ng/mL (4.21–8.18).

**Conclusions:**

These data support use of a urinary tenofovir threshold of <1500 ng/mL (tenofovir disoproxil fumarate-based regimens) as a marker of three or more missed doses for a POCT platform. However, due to low dolutegravir concentrations in urine, POCT would be limited to a readout of recent dolutegravir intake (one missed dose).

## Introduction

The success of ART and pre-exposure prophylaxis (PrEP) is dependent on individuals adhering to their medication to ensure that drug concentrations remain above clinically defined therapeutic thresholds. In routine clinical practice, point-of-care testing (POCT) yielding an immediate result would aid adherence monitoring of people receiving ART or PrEP. However, concentration ‘cut-offs’ need to be established before such devices can be clinically utilized. Since both tenofovir disoproxil fumarate and tenofovir alafenamide given together with emtricitabine or lamivudine are represented in a majority of regimens, they represent ideal targets for any POCT of adherence.

A number of matrices have been evaluated as potential markers of tenofovir disoproxil fumarate intake, including plasma—a marker of recent adherence due to tenofovir’s short plasma half-life (17 h), and dried blood spots, which serve as a measure of cumulative adherence due to the prolonged half-life of the active tenofovir diphosphate (∼17 days) in the cellular components of whole blood .^1^ Similarly, objective drug measurements from hair can provide an estimate of average adherence over weeks to months, with 1 cm of hair indicative of 1 month of drug ingestion.^2^ Furthermore, different sections of hair can help establish an individual’s adherence pattern over the preceding months, including around the time of potential seroconversion on PrEP.^3^ While cumulative measurements are required to establish long-term adherence patterns, short-term adherence metrics derived from plasma and urine can be used to quickly detect any recent changes in an individual’s behaviour and drug intake, and are more suitable for POCT due to their ease of processing.

Urine is a viable matrix for POCT as collection is non-invasive and spot urines can be self-collected. The decay profile of tenofovir and emtricitabine in urine following missed doses have been characterized to identify potential adherence cut-offs for POCT, and to establish how urine levels relate to therapeutic targets in plasma.^4,5^ The nucleoside analogues are extensively excreted in urine with tenofovir (∼80%) and emtricitabine (86%) being excreted unchanged by glomerular filtration and active tubular secretion.^6,7^ With tenofovir disoproxil fumarate dosing, urinary tenofovir concentrations were >100-fold higher than plasma and persisted longer (>7 days) following drug cessation.^6^ Urine tenofovir levels are ∼70% lower in individuals taking tenofovir alafenamide,^8^ but data are lacking on how urinary tenofovir levels compare between patients taking tenofovir disoproxil fumarate versus tenofovir alafenamide over the time course of stopping ART. Similarly, limited data are available on the urinary exposures of emtricitabine and lamivudine.^5^

Low-cost POCT devices to measure drug concentrations in patients receiving ART are currently being developed and tested.^9–11^ An antibody-based lateral flow device (UTRA) to detect tenofovir in urine has recently been co-developed by the University of California San Francisco and Abbott Diagnostics. This assay has a urine tenofovir cut-off of 1500 ng/mL and was proven to be both sensitive and specific (97%–99%) when validated against a gold standard LC-MS method.^9^ The rapid test is currently being used to support adherence monitoring in individuals taking PrEP.^12^

Here we report the pharmacokinetics of tenofovir [dosed as tenofovir disoproxil fumarate or tenofovir alafenamide], emtricitabine, lamivudine and dolutegravir in plasma and urine following drug cessation to identify short-term adherence ‘benchmarks’ for POCT.

## Materials and methods

### Study design

APT-POCT-01 (ClinicalTrials.gov identifier NCT04302896) is an open-label study assessing the pharmacokinetic profile of commonly prescribed antiretrovirals in plasma and urine over 14 days following drug intake cessation in HIV-negative healthy volunteers dosed to steady-state. Participants were recruited at the Magee-Women’s Hospital Clinical Translational Research Centre (MWH CTRC) between August 2020 and September 2021. Individuals ≥18 years of age were eligible if they were HIV-1 seronegative at screening, were in general good health in the opinion of the investigator and, if female, were willing to use an effective method of contraception (IUD, hormonal contraceptives) throughout the study duration. Participants were excluded if they were pregnant, breastfeeding, or had been using PrEP within the last 3 months.

### Study procedures

Thirty participants were randomized (1:1) to one of two treatment arms, with each arm containing 15 participants. Arm 1 participants received dolutegravir 50 mg (Tivicay^®^; ViiV Healthcare BV) in combination with emtricitabine 200 mg/tenofovir alafenamide 25 mg (Descovy^®^; Gilead Sciences Ltd) once daily for 15 days. Arm 2 received dolutegravir 50 mg (Tivicay^®^; ViiV Healthcare BV) in combination with tenofovir disoproxil fumarate 300 mg (Viread^®^; Gilead Sciences Ltd) and lamivudine 300 mg (Apotex, FL, USA) once daily for 15 days. Doses were directly observed or recorded by participants to monitor adherence. Plasma and spot urine samples were collected during the ‘dosing phase’, on Day 1, 2 and 8, and after ingestion of the final dose on Day 15, at 0, 1, 4, 24, 48, 72, 96, 168 and 336 h post-dose during the ‘drug cessation phase’.

### Sample collection

Specimens were collected and processed at MWH CTRC. Whole blood was collected in K_2_EDTA tubes, centrifuged (1500×**g** 10 min at 4°C) and the plasma aliquoted. Spot urine samples were collected mid-stream using a urine collection cup and kept at room temperature until transported to the laboratory. Upon gentle mixing, urine was aliquoted into cryovials. All samples were stored at −70°C.

### Drug quantification

Drug concentrations in plasma and urine were measured using validated LC-MS assays. The calibration curves for the nucleoside analogues ranged between 0.5 and 500 ng/mL (tenofovir alafenamide), 1 and 1000 ng/mL (tenofovir), and 5 and 5000 ng/mL (emtricitabine/lamivudine) in plasma, and between 12.5 and 5000 ng/mL in urine. Dolutegravir calibration curve ranges were 10–10 000 ng/mL (plasma) and 10–4000 ng/mL (urine). All methods were validated in accordance with the FDA guidelines for Bioanalytical Method Validation.^13^ Urine samples from the study participants were diluted 1:1, 1:10 and 1:100 in order to achieve concentrations within the assay calibration range and over the course of the sampling period.

### Pharmacokinetic analysis

Drug concentrations in plasma, urine and saliva were presented using descriptive statistics. Pre-dose samples taken at Day 1 that were below the assay lower limit of quantification (LLQ) were set as 0 ng/mL. Post-dose samples that were <LLQ were assigned half-LLQ values, and for consecutive post-dose samples <LLQ, the first was assigned a half-LLQ value and any subsequent samples were excluded. Pharmacokinetic parameters including terminal elimination half-life (*t*_½_), *C*_max_, *T*_max_, *C*_min_, AUC over the dosing interval (AUC_24_) and to the last measurable time point (AUC_last_) were calculated using non-compartmental methods (Phoenix 64, WinNonlin, version 8.3).

Drug concentrations were log-transformed and a Pearson correlation coefficient (r) used to estimate the correlation between quantifiable concentrations and exposures (AUC) in urine and plasma (SPSS version 29.0.1.0).

Non-linear mixed-effects models (NONMEM v.7.4) with Laplacian estimation were applied to determine drug disposition between matrices and relationship with systemic thresholds—dolutegravir time above protein-adjusted IC_90_ [PA-IC_90_ (64 ng/mL)], recommended minimum effective concentration (MEC; 324 ng/mL)^14^ and the concentration of tenofovir in urine indicative of more than one missed dose (1500 ng/mL, tenofovir disoproxil fumarate).^11^ A 300 mg tablet of tenofovir disoproxil fumarate is known to contain 136 mg of tenofovir; the latter was used as the input dose for the model. The modelling process was executed and documented using Pirana (v. 2.9.0) interfaced with NONMEM and tenofovir and dolutegravir concentrations below assay LLQs were included using the M3 method (determining the probability of a sample being below the LLQ).^15–17^ Model evaluation in the form of visual predictive check was performed using Perl-Speaks-NONMEM (PsN; v. 3.4.2) and resultant plots generated with Xpose4 in RStudio (v. 2023.06.02).^18,19^

### Ethics

The study was conducted in accordance with the Declaration of Helsinki and applicable local and US regulatory requirements. Participants were asked to provide written informed consent and the study protocol and consent documents were approved by the University of Pittsburgh Institutional Review Board (CR19100009-005).

## Results

A total of 29 patient pharmacokinetic profiles were evaluated: 15 subjects (Arm 1) and 14 subjects (Arm 2). One participant (Arm 2) withdrew from the study and was excluded. Among the 29 participants, 21 (72%) were female at birth and 26 (90%) were Caucasian. Participant demographics are shown in Table 1. There were no substantial differences in mean clinical measures between the two study arms. Participants in Arm 1 had significantly lower creatinine levels than those in Arm 2, potentially attributed to the higher proportion of females in this group (Arm 1 = 87% female; Arm 2 = 57% female).

**Table 1.** Participant demographics and clinical characteristics (*n* = 29)

Parameter	Arm 1 (TAF/FTC/DTG)	Arm 2 (TDF/3TC/DTG)	Total	*P* value^a^
*N* (%)	15 (51.7)	14 (48.3)	29 (100)	
Age (years)	34 (21–58)	38 (20–67)	36 (20–67)	0.948
Weight (kg)	77.1 (56.2–97.5)	68.0 (49.0–97.5)	73.0 (49.0–97.5)	0.407
Height (cm)	165.1 (152.4–185.4)	165.1 (142.2–185.4)	165.1 (142.2–185.4)	0.878
BMI (kg/m^2^)	29.5 (20.2–36.5)	25.9 (20.4–29.9)	26.7 (20.2–36.5)	0.138
Sex at birth, *n* (%)				
Female	13 (86.7)^b^	8 (57.1)	21 (72.4)	0.075
Male	2 (13.3)	6 (42.9)	8 (27.6)	
Ethnicity				
White	12 (80.0)	13 (92.9)	25 (86.2)	0.316
Black African-American	1 (6.7)	0 (0.0)	1 (3.4)	
Asian	2 (13.3)	1 (7.1)	3 (10.3)	
Creatinine (mg/dL)	0.76 (0.59–1.10)	0.87 (0.69–1.11)	0.80 (0.59–1.11)	0.029
Bilirubin (mg/dL)	0.50 (0.30–0.70)	0.55 (0.30–1.60)	0.50 (0.30–1.60)	0.168
ALT (U/L)	13.0 (9.0–49.0)	13.5 (8.0–34.0	13.0 (8.0–49.0)	0.599
AST (U/L)	15.0 (10.0–24.0)	17.5 (10.0–21.0)	17.0 (10.0–24.0)	0.417

Data are expressed as median (range) unless otherwise stated. TAF, tenofovir alafenamide; TDF, tenofovir disoproxil fumarate; FTC, emtricitabine; 3TC, lamivudine; DTG, dolutegravir.

^a^Differences between study arms analysed by Mann–Whitney *U*-test for continuous data and χ^2^ test for categorical data.

^b^One participant identified as gender-non-conforming.

**Figure 1.**
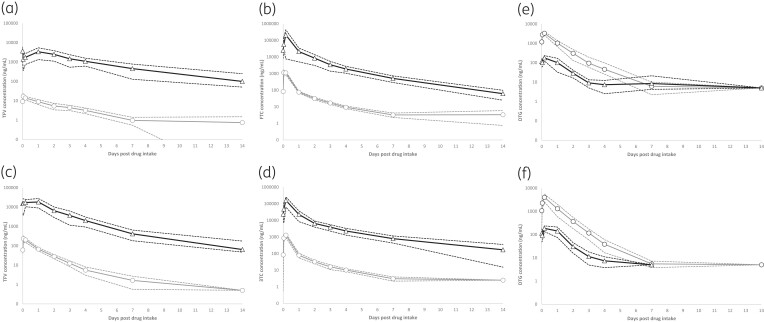
Geometric mean (95% CI—dashed lines) concentrations in plasma and urine for (a) tenofovir (tenofovir alafenamide; TFV_TAF_), (b) emtricitabine (FTC), (c) tenofovir (tenofovir disoproxil; TFV_TDF_), (d) lamivudine (3TC) and (e) dolutegravir (DTG) (Arm 1) and (f) DTG (Arm 2), during the 14 day drug intake cessation period. Plasma values are indicated with grey lines (circles) and urine values with black lines (triangles). Detectable concentrations below the assay LLQ are expressed as half-LLQ values.

Geometric mean (95% CI) concentrations in plasma and urine for the NRTI and dolutegravir during the 14 day cessation period are shown in Figure 1 and the pharmacokinetic parameters for all analytes and compartments are summarized in Table 2.

**Table 2.** Geometric mean (95% CI) pharmacokinetic parameters for tenofovir, emtricitabine (Arm 1), tenofovir, lamivudine (Arm 2) and dolutegravir (both arms) in plasma and urine after 14 days drug intake cessation

Parameter	Arm 1 (TAF/FTC/DTG) (*n* = 15)			Arm 2 (TDF/3TC/DTG) (*n* = 14)		
	TFV_TAF_	FTC	DTG	TFV_TDF_	3TC	DTG
Plasma						
*C*_max_ (ng/mL)	20.2 (15.9–25.6)	1837 (1500–2250)	3651 (29 814 470)	287 (214–385)	1496 (1138–1965)	4255 (3145–5756)
*T*_max_ (h)	1.45 (1.02–2.06)	1.59 (1.09–2.31)	2.52 (1.73–3.66)	1.81 (1.20–2.73)	2.44 (1.64–3.63)	3.73 (2.44–5.69)
*C*_24_ (ng/mL)	8.90 (6.38–11.4)	75.7 (66.7–84.6)	1032 (670–1394)	67.0 (54.6–79.4)	78.9 (55.0–103)	1297 (590–2004)
AUC_last_ (ng·h/mL)	889 (584–1354)	20 591 (17 505–24 221)	82 885 (63 818–107 648)	5979 (4776–7486)	21 015 (15 990–27 619)	97 029 (68 661–137 118)
*T*_last_ (h)	234 (173–318)	202 (170–241)	174 (143–210)	278 (221–350)	183 (146–231)	170 (147–196)
*t*_½_ (h)^a^	38.0 (29.6, 48.7)	43.9 (34.7–55.7)	22.6 (17.7–29.0)	27.0 (20.6–35.3)	42.1 (31.3–56.6)	19.9 (17.3–22.9)
Urine						
*C*_max_ (ng/mL)	5515 (3858–7883)	247 833 (143 664–427 535)	274 (195–383)	33 410 (23 011–48 510)	184 805 (121 531–281 023)	239 (165–344)
*T*_max_ (h)	0.00 (0.00–24.0)	4.00 (0.00–4.00)	4.00 (0.00–24.0)	2.50 (0.00–24.0)	4.00 (0.00–4.00)	0.00 (4.00–24.0)
*C*_24_ (ng/mL)	3389 (1334–5444)	21 130 (7648–34 613)	120 (−49.9 to 291)	18 174 (8909–27 440)	22 588 (8310–36 866)	142 (74.8–210)
AUC_last_ (ng·h/mL)	358 893 (251 510–512 123)	4 299 487 (2 771 343–6 670 263)	7251 (4610–11 405)	1 171 405 (840 804–1 631 997)	3 389 442 (2 301 342–4 992 009)	7274 (5088–10 401)
*T*_last_ (h)	336 (336–336)	336 (336–336)	95.7 (73.5–125)	336 (336–336)	336 (336–336)	92.9 (74.8–115)
*t*_½_ (h)^a^	66.1 (54.2–80.6)	46.5 (40.3–53.6)	20.6 (13.7–31.0)	49.3 (40.4–60.3)	51.5 (41.6–63.7)	17.5 (12.6–24.1)

TAF, tenofovir alafenamide; TDF, tenofovir disoproxil; FTC, emtricitabine; 3TC, lamivudine; DTG, dolutegravir.

^a^Terminal elimination half-life to the last measurable concentration (*T*_last_).

### NRTI pharmacokinetics

**Figure 2.**
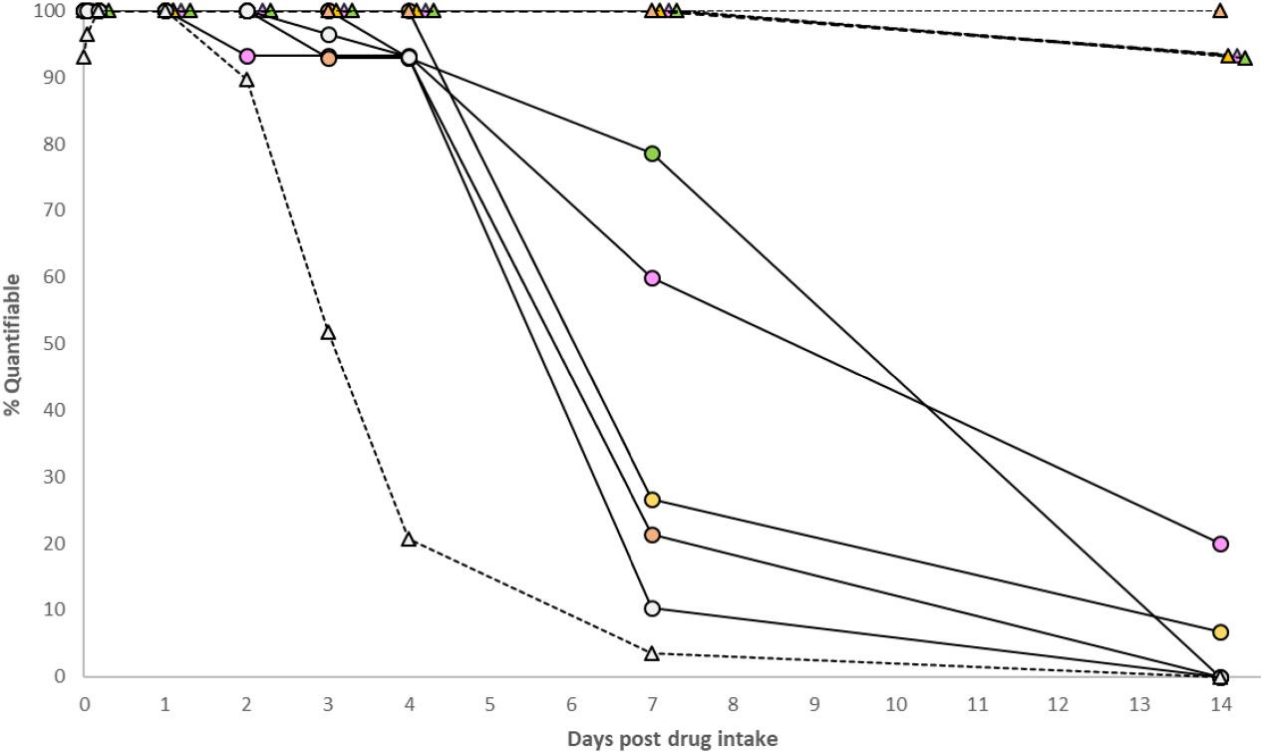
Proportion of plasma and urine samples with NRTI [tenofovir alafenamide (TFV_TAF_), tenofovir disoproxil fumarate (TFV_TDF_), emtricitabine (FTC), lamivudine (3TC)] and dolutegravir (DTG) levels above the LC-MS assay LLQ. Plasma values are indicated with a solid line (circles) and urine values with a dotted line (triangles). TFV_TAF _= pink; TFV_TDF_ = green; FTC = yellow; 3TC = orange; DTG = grey. The LLQs were 1 ng/mL (TFV), 5 ng/mL (FTC/3TC) and 10 ng/mL (DTG) in plasma, and 12.5 ng/mL (NRTI) and 10 ng/mL (DTG) in urine. This figure appears in colour in the online version of *JAC* and in black and white in the print version of *JAC*.

Steady-state NRTI trough concentrations (*C*_24_) in plasma (Table 2) were consistent with trough levels previously reported [geometric mean (range) tenofovir disoproxil fumarate 50 ng/mL (35–77); tenofovir alafenamide 1–10 ng/mL; emtricitabine 75 ng/mL (55–104); lamivudine 40 ng/mL (20–110)].^20–23^ Measured [geometric mean (range)] tenofovir urine concentrations on Day 15, between 0 and 24 h post-dose, were 194 (81–400)-fold (tenofovir alafenamide) and 139 (64–271)-fold (tenofovir disoproxil fumarate) higher than steady-state plasma levels, rising to 355 (198–492)-fold and 254 (168–358)-fold over 48–336 h post intake cessation. Tenofovir elimination from urine was more prolonged compared with plasma (Table 2). All spot urine tenofovir concentrations were quantifiable up to 7 days post-dose irrespective of the formulation administered (Figure 2).

Steady-state tenofovir plasma and urine concentrations were ∼80% lower in participants receiving tenofovir alafenamide, compared with those receiving tenofovir disoproxil fumarate (Figure 1). However, the difference in tenofovir exposures between the two formulations diminished over the course of the intake cessation period. Tenofovir alafenamide itself was only quantifiable between 1 and 4 h post-dose [geometric mean (range) plasma 151 ng/mL (66–236); urine 570 ng/mL (27.3–5590] with undetectable levels at all subsequent timepoints.

Tenofovir urine concentrations exceeded the cut-off of 1500 ng/mL at 24 and 48 h post intake cessation in 93% and 86% of the participants who received tenofovir disoproxil. At 96 h (4 days), the number above this cut-off fell to 64%. In the tenofovir alafenamide arm, the majority (>80%) had urinary tenofovir levels above this cut-off at 24–48 h post-dose, but the proportion above target fell to 47% after 4 days.

Emtricitabine and lamivudine levels in urine were ∼200-fold higher than absolute levels in plasma and were eliminated in a biphasic manner. Urinary concentrations peaked at 4 h (*C*_max_ ∼200 µg/ml) and decayed rapidly (∼1 log) over 24 h, followed by a more gradual decline during the cessation period. The *t*_½_ values, calculated to the last measurable concentration, were comparable in urine and plasma (Table 2). Geometric mean (95% CI) emtricitabine and lamivudine urine concentrations at 96 h post-dose, used as a marker of three consecutive missed doses, were 1892 (1054–2731) and 2277 ng/mL (1284–3270), respectively. At this timepoint, corresponding plasma levels for both entities were <10 ng/mL.

### Dolutegravir pharmacokinetics

Dolutegravir concentrations in urine (*C*_24_ = 130 ng/mL) equated to only 7% and 18% of plasma at steady-state and over the intake cessation phase. Plasma concentrations remained above the assay quantification limit in 93% of subjects at 4 days post-intake cessation (42 ng/mL), but were quantifiable in urine in only six subjects (21%) at this timepoint. All urine samples were undetectable beyond 7 days post-dose (Figure 1). Dolutegravir elimination was biphasic and was equivalent in the plasma and urine compartments (*t*_½_ ∼20 h).

### Correlations

Measurable urine and plasma concentrations over the steady-state and drug cessation period were strongly correlated for dolutegravir, emtricitabine, lamivudine and tenofovir dosed as tenofovir disoproxil fumarate (r^2^ ≥ 0.75), but only a weak to moderate relationship (r^2^ = 0.36) was observed for tenofovir when dosed as tenofovir alafenamide. Correlations using drug exposures (AUC_24_, AUC_72_, AUC_last_), performed to mitigate the effect of repeated measures, showed comparable results (Table S1, available as Supplementary data at *JAC* Online).

### Predicted concentrations

Plasma dolutegravir and tenofovir (tenofovir disoproxil fumarate) were described by two-compartment models; model parameters are summarized in Table S2, and visual predictive checks in Figure S1. Urine concentrations were modelled as a proportion of plasma using an accumulation ratio as a proportionality constant (ARU). ARU (90% CI) was 0.086 (0.0815–0.994) for dolutegravir and 244 (192–313) for tenofovir, with interindividual variabilities (90% CI) of 47.4% (35.0%–54.4%) and 53.3% (24.4%–71.0%), respectively. Covariates were not assessed.

Predicted concentrations in plasma and urine following one to three missed doses are summarized in Table 3. The median (range) predicted time to reach the plasma dolutegravir PA-IC_90_ and MEC was 83.5 h (41.0–152) and 49.0 h (23.7–78.9). At these timepoints, the corresponding predicted geometric mean (90% CI) dolutegravir urine concentrations were 5.42 (4.37–6.46) and 27.4 ng/mL (22.1–32.7), respectively. At 48 h post-dose, which corresponds to a single missed dose, plasma dolutegravir levels were in range of the MEC and predicted urine levels were 29.5 ng/mL; however, after two or more missed doses predicted dolutegravir urine levels were <10 ng/mL. By contrast, tenofovir (administered as tenofovir disoproxil) in urine remained high after one to three missed doses, with a urine concentration of 1565 ng/mL (975–2155) indicative of three missed doses. When tenofovir urine concentrations reached the defined threshold of 1500 ng/mL at 101 h (58.6–205) [4.2 days (2.4–8.5)] the equivalent predicted concentration in plasma was 6.20 ng/mL (4.21–8.18).

**Table 3.** Model-predicted geometric mean (90% CI) dolutegravir (DTG) and tenofovir disoproxil fumarate (TFV_TDF_) concentrations in plasma and urine in healthy volunteers following drug intake cessation (1–3 missed doses)

	Number of missed doses (hours post-intake cessation)			Threshold	
DTG	1 (48)	2 (72)	3 (96)	MEC_plasma_	PA-IC_90_
Plasma, ng/mL	348 (248–449)	104 (60.8–146)	34.1 (14.1–54.2)	**324**	**64.0**
Urine, ng/mL	29.5 (5.50–53.5)	8.77 (−2.73 to 20.3)	2.89 (−2.68 to 8.46)	27.4 (22.1–32.7)	5.42 (4.37–6.46)
TFV_TDF_	1 (48)	2 (72)	3 (96)	Urine concentration after 1 missed dose	
Plasma, ng/mL	26.6 (23.1–30.1)	11.8 (9.84–7.80)	6.46 (5.12–7.80)	6.20 (4.21–8.18)	
Urine, ng/mL	6441 (4340–8542)	2859 (1850–3868)	1565 (975–2155)	**1500**	

Bold values indicate the threshold/cut-off concentrations; all other values are model-predicted.

## Discussion

Our data indicate that unchanged drug in urine can serve as a potential surrogate marker of systemic exposures and medication adherence, since NRTI and dolutegravir concentrations in urine were correlated with plasma over the course of the dosing interval and after stopping treatment for 14 days.

Tenofovir concentrations in urine remained high for several days and persisted longer than corresponding levels in plasma, reinforcing the fact that spot urine samples are suitable for objectively determining recent adherence.

The lower (∼80%) tenofovir urinary concentrations seen with tenofovir alafenamide are a direct consequence of the prodrug’s unique biotransformation pathway. While tenofovir disoproxil fumarate is metabolized directly to tenofovir in the plasma and gut, tenofovir alafenamide is converted to tenofovir intracellularly in PBMCs, leading to reduced tenofovir loading in plasma.^24^ Tenofovir alafenamide itself is not a useful metric; levels of the prodrug were only detected in urine at steady-state during the dosing interval (1–4 h) and were undetectable beyond 24 h post-dose. Tenofovir alafenamide primarily undergoes hepatobiliary clearance, with the majority converted to tenofovir and less than 1% eliminated unchanged in urine.^25^

To the best of our knowledge, this is the first study to present data on emtricitabine and lamivudine in urine following simulated missed doses. Emtricitabine and lamivudine urinary concentrations were extremely high (in the µg/mL range), and therefore may be more easily measured using urine-based POCT. Although urine emtricitabine and lamivudine concentrations declined substantially between 4 and 24 h, both agents exhibited a slower decay phase in urine over the intake cessation period which will, in turn, permit detection using POCT in individuals with suboptimal or erratic adherence.

As expected, dolutegravir concentrations in urine were low and accounted for less than 10% of circulating plasma concentrations in this study. Using the population pharmacokinetics model, we were able to establish that when levels of dolutegravir reached its PA-IC_90_ (≤64 ng/mL), concentrations in urine were below the LLQ of the LC-MS assay (<10 ng/mL). Dolutegravir is primarily eliminated via faeces, with urinary elimination accounting for only 31% of an administered dose, and of this, <1% is eliminated as unchanged drug in urine. Consequently, a highly sensitive POCT is needed to detect dolutegravir urine levels that are indicative of subtherapeutic systemic exposures, and the utility of a urine-based POCT would be limited to a readout of recent drug intake within the last 48 h or after a single missed dose. However, given that an inactive ether glucuronide, formed via UGT1A1, accounts for approximately 19% of the dose eliminated in urine,^26^ POCT that does not discriminate between the parent and the glucuronidated form may offer enhanced sensitivity. Further investigations are therefore warranted to investigate the decay kinetics of the dolutegravir glucuronide in urine in the context of missed doses.

The drug intake cessation data from this study can be used to inform a threshold that has a high likelihood of non-adherence. POCT used to detect non-adherence should be tuned to have a high negative predictive value. In other words, if the test result is negative there is a very high certainty of non-adherence, and there is a reduced likelihood of a false-negative result, that is, an adherent patient being incorrectly classified as non-adherent, which could cause the patient unnecessary distress.^27^

Only 36% of individuals receiving tenofovir disoproxil fumarate exhibited tenofovir urine levels below 1500 ng/mL at 4 days post-dose (equivalent to three missed doses), suggesting that POCT with a cut-off of 1500 ng/mL would have relatively low sensitivity, and detect non-adherence in a minority of cases in this small cohort. However, reduced test sensitivity in identifying non-adherence is justifiable in order to prevent potential misclassification of fully adherent subjects. This underscores the importance of prioritizing specificity over sensitivity to accurately identify non-adherent individuals and avoid erroneous classification of those who are fully adherent. Based on the model-predicted concentrations, a tenofovir (disoproxil fumarate) urine concentration of 975 ng/mL (lower bound 90% CI after three simulated missed doses; Table 3) could indicate a point where, at least within this cohort, the misclassification of adherent individuals and the probability of false negatives from POCT are minimized.

**Figure 3.**
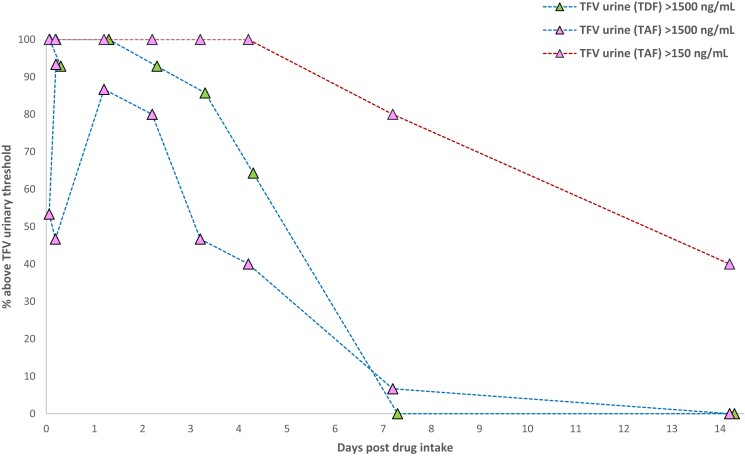
Proportion of urine samples with tenofovir (TFV) concentrations >1500 ng/mL [tenofovir disoproxil fumarate (TDF) and tenofovir alafenamide (TAF) dosing] or >150 ng/mL (TAF dosing). The blue dashed line shows the 1500 ng/mL threshold and the red dashed line shows the 150 ng/mL threshold. TFV_TAF_ = pink triangles; TFV_TDF_ = green triangles. This figure appears in colour in the online version of *JAC* and in black and white in the print version of *JAC*.

Based on the ∼8-fold lower urinary tenofovir exposures anticipated with tenofovir alafenamide dosing, a urinary cut-off of 1500 ng/mL could increase the likelihood of false-negative POCT readouts for those receiving tenofovir alafenamide, that is, adherent individuals being incorrectly identified as non-adherent.^28^ Sevenler and colleagues^29^ used a more conservative threshold of 150 ng/mL to validate the performance of their lateral flow immunoassay, but this adjusted threshold is yet to be formally validated in individuals receiving tenofovir alafenamide. Interestingly, for participants dosed with tenofovir alafenamide, we observed a more prolonged elimination of tenofovir from the urine, as compared with the disoproxil regimen. As a consequence, tenofovir (alafenamide) urine levels remained above 150 ng/mL in all participants at 4 days (three consecutive missed doses) and a significant proportion of subjects (∼40%) remained above even after 14 days of intake cessation (Figure 3). POCT with an adjusted cut-off of 150 ng/ml would therefore have very low sensitivity but high certainty of detecting non-adherence as, based on these data, individuals would need to miss over a week’s worth of doses before tenofovir urine levels fall below this target. The measured tenofovir urinary concentration at the lower bound of the 95% CI after three simulated missed doses of tenofovir alafenamide was 616 ng/mL (Figure 1), suggesting that a potential benchmark to detect non-adherence, whilst minimizing misclassification of truly adherent individuals, may lie somewhere between the two previously proposed cut-offs. UrSure Inc. have developed a point-of-care lateral flow immunoassay with a tenofovir cut-off of 650 ng/mL, although this has not yet been tested for tenofovir alafenamide adherence.^30^ Further work utilizing pharmacokinetic data to assess the performance of POCT with different cut-offs is needed to identify viable adherence targets for tenofovir alafenamide-based regimens.

There are several limitations to our analyses. Spot urine samples were collected, as opposed to the sampling the total volume of urine expelled over time intervals (e.g. 0–4, 8–12, 12–24 h); the latter approach accounts for emptying of the bladder over the elimination time course and enables the true elimination of drug from urine to be determined. We choose to collect spot urine samples since these more closely represent the ‘real-life’ situation of when a patient attends clinic. As urine specific gravity measurements were not taken, the spot urine concentrations reported here are potentially modulated by a subject’s fluid intake, hydration state and frequency of bladder emptying. For example, excessive fluid intake can dilute urinary drug concentrations and result in potential false-negative results. However, given that NRTI urinary concentrations are in the µg/mL range, and significantly higher than plasma concentrations, such volumetric effects are anticipated to be minimal. Population pharmacokinetics modelling was utilized in order to predict urine and plasma concentrations at relevant therapeutic thresholds. Given the strong linear relationships between tenofovir and tenofovir (disoproxil) plasma and urine concentrations, and relatively modest patient numbers, proportionality constants were used to describe the association between the plasma and urine concentrations rather than the application of transfer rate constants to and from compartments. This provided the most parsimonious models with fewer parameters and without significant difficulties converging or excessive run-times. However, some misspecification was noted for the urine peak concentrations, which may be mitigated by use of transfer rate constants and transit/delay compartments into the urine, but this further increases the number of parameters and introduces identifiability issues. Despite this, the models generally described the central tendency of the data and predictions were consistent with the literature. Similar problems were encountered whilst attempting to develop a simultaneous tenofovir (alafenamide) plasma and urine model, particularly with regard to parameter identifiability, and ultimately a population pharmacokinetics model was not pursued.

In conclusion, urinary NRTI concentrations correlate with the corresponding levels in plasma and can serve as a viable POCT specimen to determine short-term adherence among adults receiving PrEP or ART. Given the low concentrations of dolutegravir in urine, utility of a urine-based POCT would be limited to a readout of recent drug intake within 48 h (one missed dose). These pharmacokinetic data can be used to inform adherence benchmarks for validation of existing urinary POCT, and to tune future technologies.

## Supplementary Material

dkae147_Supplementary_Data

## Transparency declarations

S.K. has received research grants and travel bursaries from Gilead, ViiV, Merck, Bristol Myers Squibb, Boehringer Ingelheim and Janssen Pharmaceuticals. All other authors: none to declare. The authors have no other relevant affiliations or financial involvement with any organization or entity with a financial interest in or financial conflict with the subject matter or materials discussed in the manuscript apart from those disclosed. No writing assistance was utilized in the production of this manuscript.

## Supplementary data

Figure S1 and Tables S1 and S2 are available as Supplementary data at *JAC* Online.
