# Supplementary material for: Tenofovir, emtricitabine, lamivudine and dolutegravir concentrations in plasma and urine following drug intake cessation in a randomized controlled directly observed pharmacokinetic trial to aid point-of-care testing
Source: J Antimicrob Chemother. 2024 May 17;79(7):1597–605. doi: 10.1093/jac/dkae147 (PMC11215529; doi:10.1093/jac/dkae147)
Supplement: dkae147_Supplementary_Data [file dkae147_supplementary_data.docx]

**Supplementary Figure Legends**

**Figure S1.** Visual predictive check of **(a)** the TFV_TDF_ and **(b)** DTG model stratified by sampling matrix based on 1000 simulations. The lower and upper dashed lines are the 5th and 95th percentiles of the observations and the solid line the median. The shaded areas are the 95% CI of the 5th, 95th percentiles (light grey) and median (grey) of the simulations. Open circles are the observations. In the lower panels, observed proportions of samples below the lower limit of quantification are shown as open circles and 95% CI of the simulated proportion as the shaded area.

**Figure S1.**

**
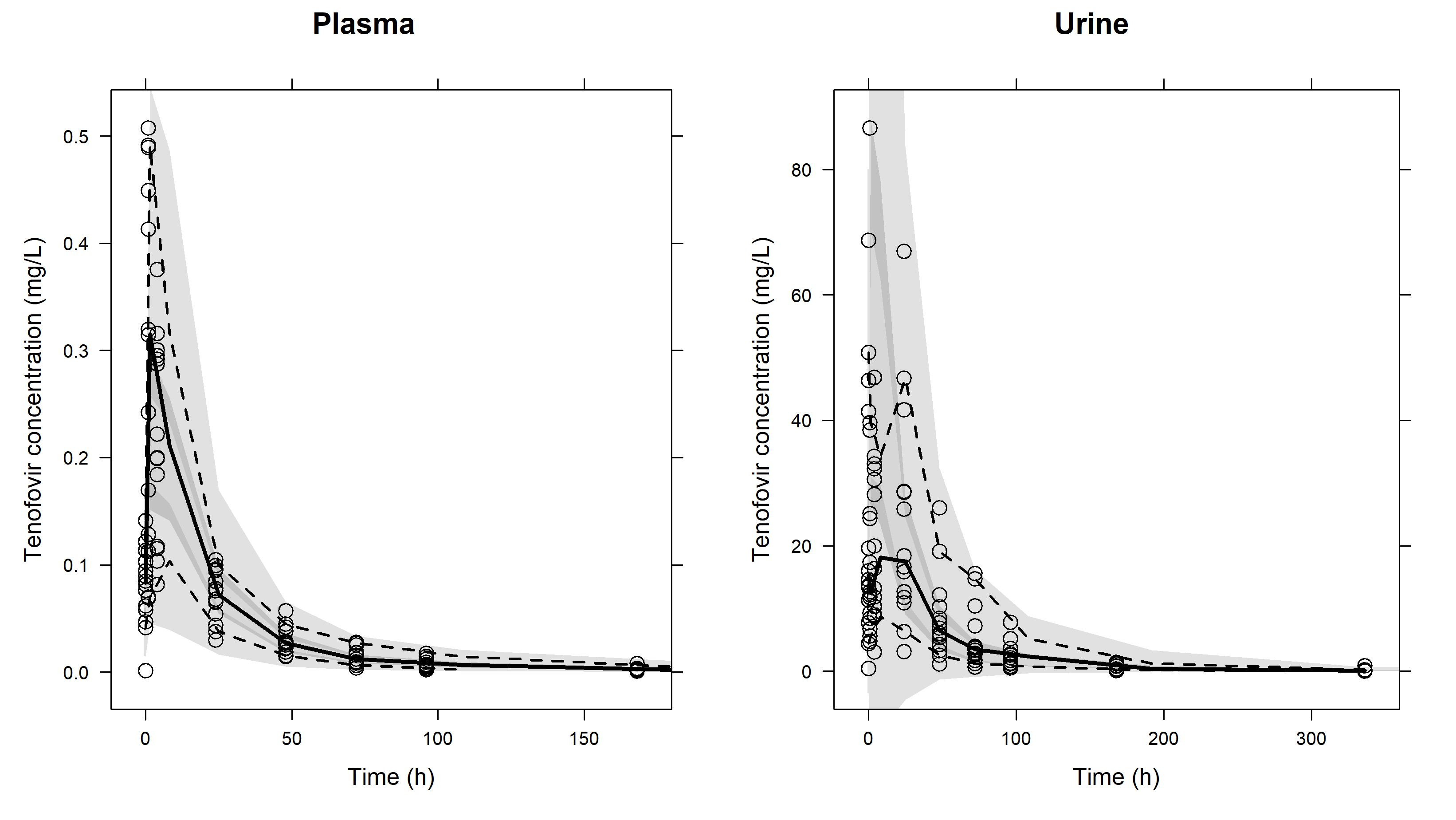
**

**a**


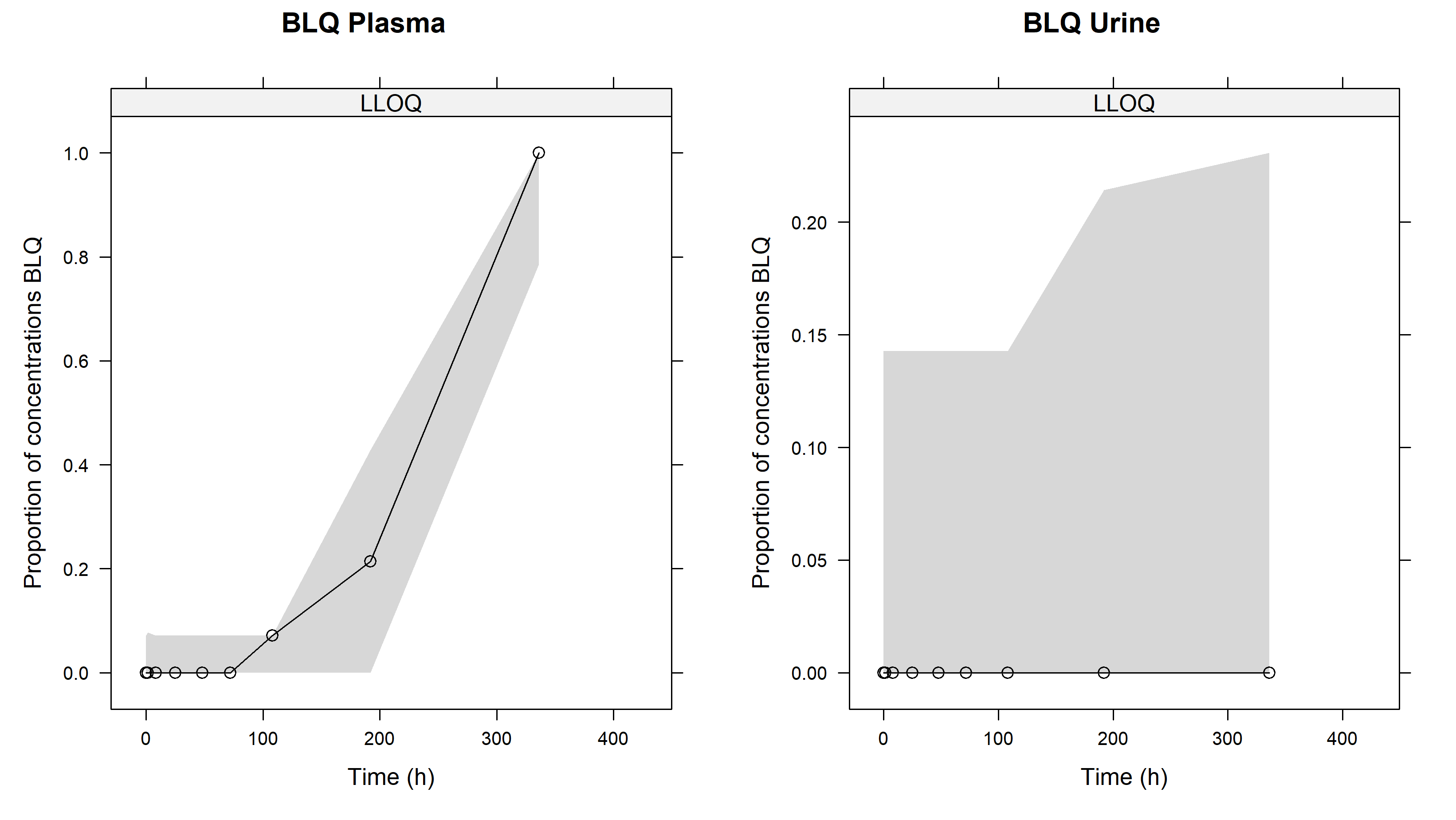

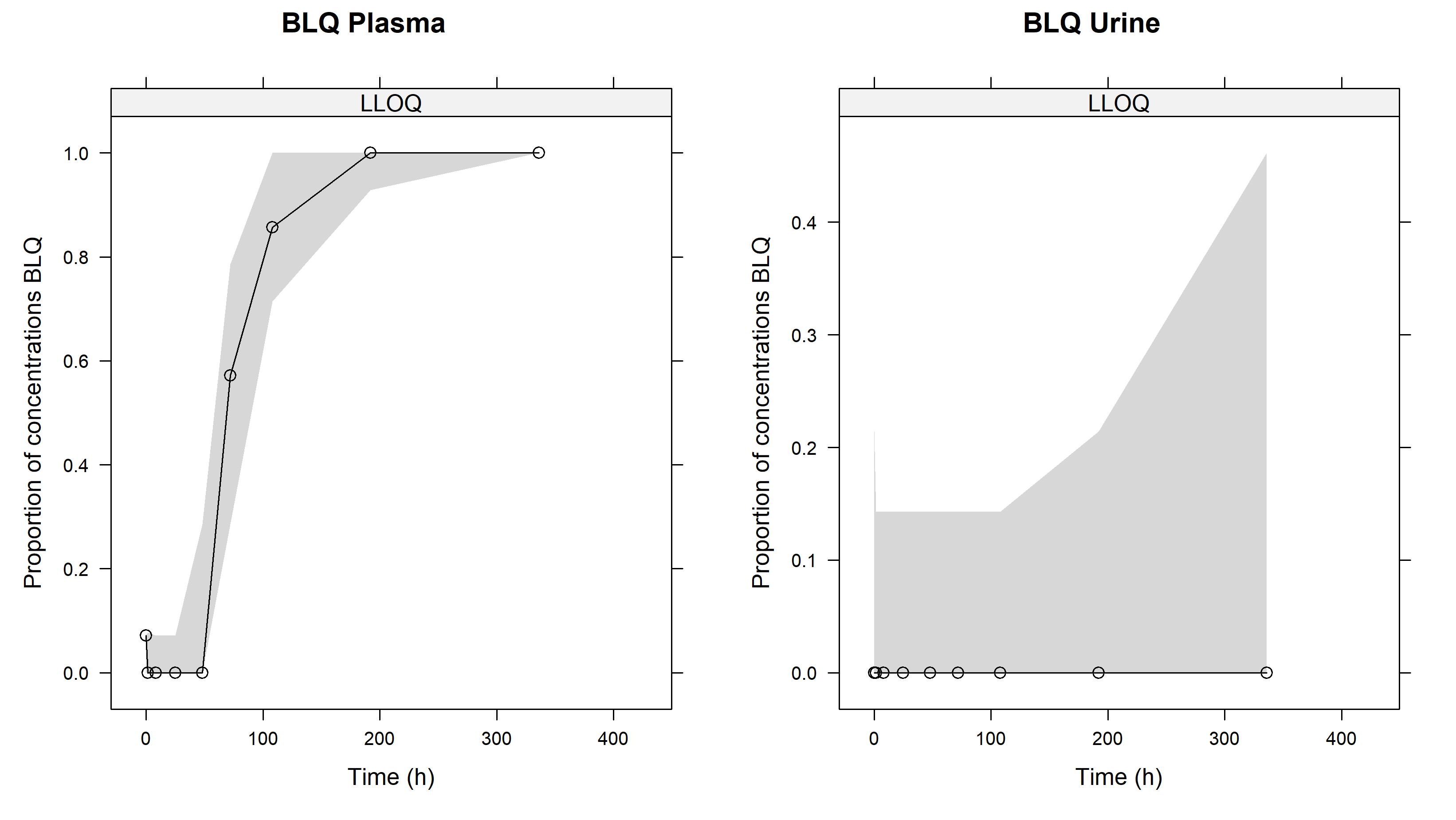


*
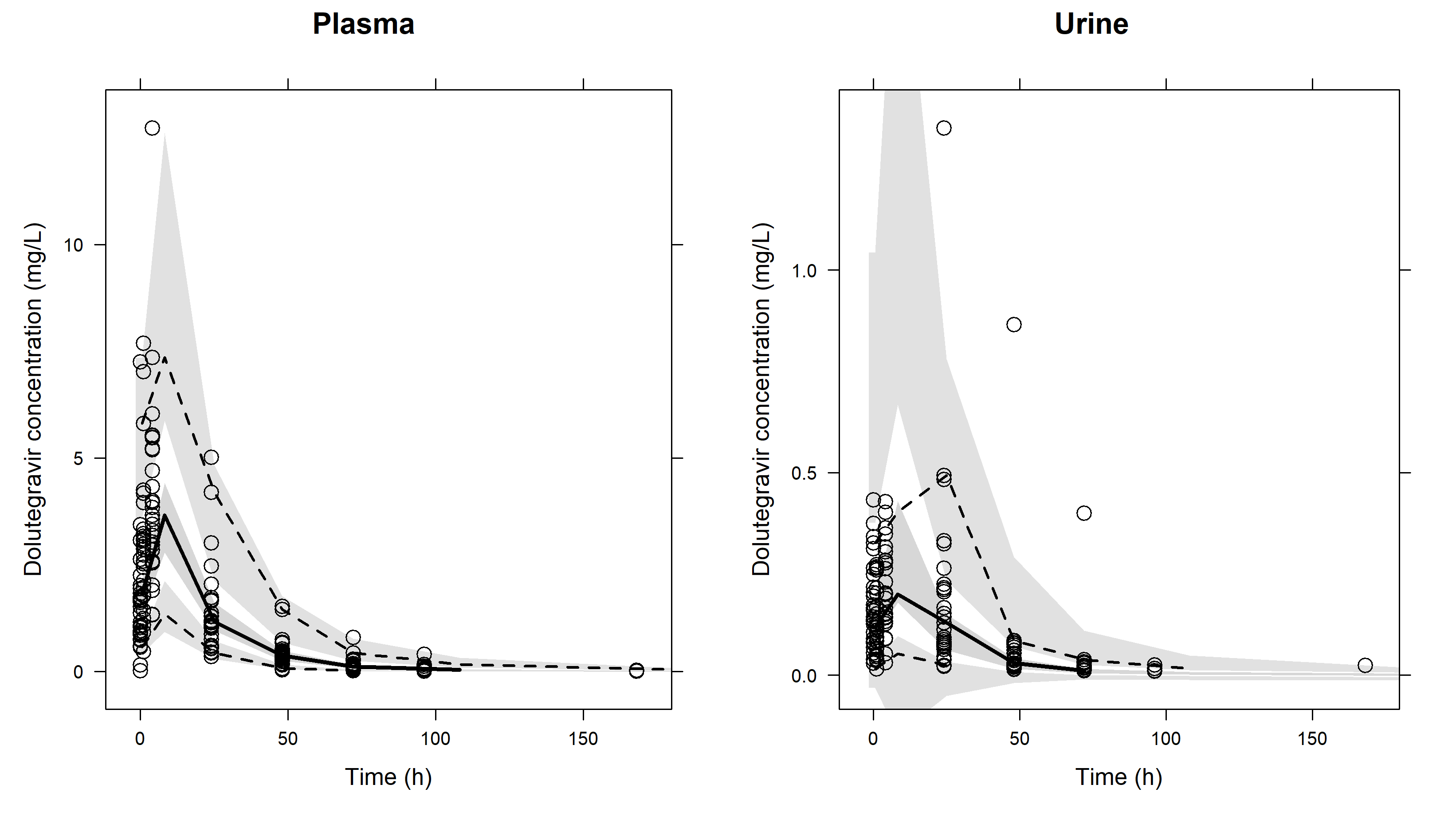
*

**b**

*
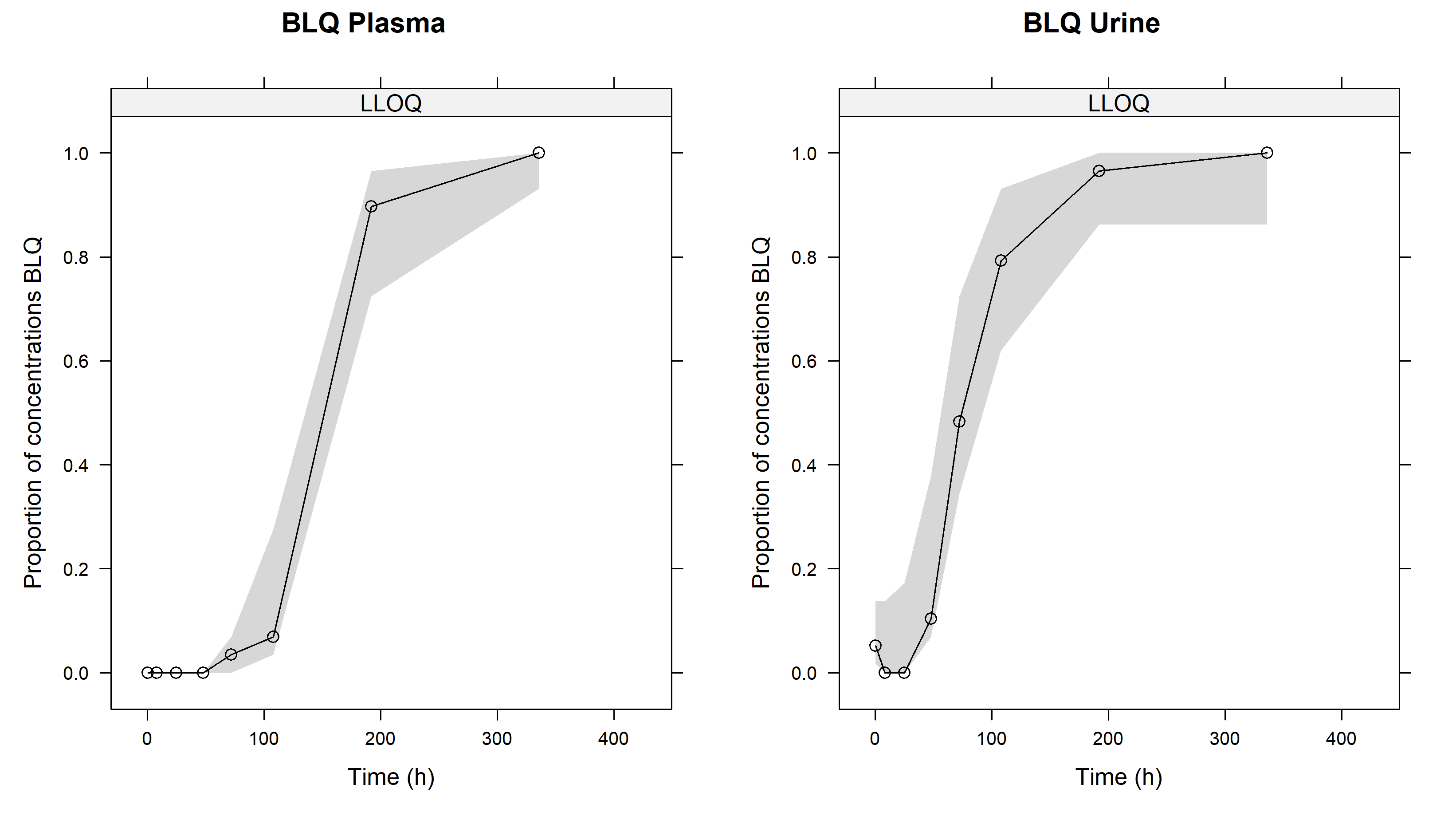
*

**Table S1.** Correlation (r; P value) of quantifiable spot urine and plasma concentrations (ng/mL) and exposures (ng.h/mL) for (A) TFV_TAF_, (B) FTC, (C) TFV_TDF_ , (D) 3TC and (E) DTG.

| **Drug (n subjects)** | **All samples (ng/mL)** | **AUC_0-last_ (ng.h/mL)** | **AUC_0-24_ (ng.h/mL)** | **AUC_0-72_ (ng.h/mL)** |
| --- | --- | --- | --- | --- |
| **TFV_TAF_ (15)** | **0.360** (<0.001) | 0.425 (0.114) | 0.338 (0.218) | 0.401 (0.138) |
| **FTC (15)** | **0.819** (<0.001) | 0.479 (0.074) | **0.573** (0.025) | **0.513** (0.050) |
| **TFV_TDF_ (14)** | **0.745** (<0.001) | **0.536** (0.048) | **0.607** (0.021) | **0.530** (0.050) |
| **3TC (14)** | **0.818** (<0.001) | **0.752** (0.002) | **0.719** (0.004) | **0.723** (0.003) |
| **DTG (29)** | **0.779** (<0.001) | **0.727** (<0.001) | **0.718** (<0.001) | **0.677** (<0.001) |

**Table S2.** Tenofovir (from TDF) and dolutegravir population pharmacokinetics model parameters and 90% CI developed from plasma and urine concentration-time data in healthy volunteers following drug intake cessation

|  | **TFV** | | **DTG** | |
| --- | --- | --- | --- | --- |
| **Parameter** | **Estimate** | **90% CI^1^** | **Estimate** | **90% CI^1^** |
| *Fixed effects* |  |  |  |  |
| CL/F (L/h) | 40.7 | 35.0-47.2 | 0.867 | 0.814-0.972 |
| V_c_/F (L) | 839 | 675-1062 | 15.9 | 14.4-17.9 |
| Q/F (L/h) | 2.67 | 1.33-4.12 | 0.0144 | 0.0114-0.0362 |
| V_p_/F (L) | 197 | 122-283 | 0.381 | 0.381-0.830 |
| k_a_ (h^-1^) | 3 fixed | - | 1.88 | 1.82-8.44 |
| Absorption lag-time (h) | - | - | 0.755 | 0.755-0.956 |
| ARU | 244 | 192-313 | 0.0862 | 0.0815-0.994 |
| *Random effects* |  |  |  |  |
| IIV CL/F (%) | 28.8 | 19.9-35.1 | 49.1 | 34.6-49.0 |
| IIV V_c_/F (%) | 39.0 | 22.9-53.8 | 50.9 | 32.7-51.4 |
| IIV k_a_ (%) | - | - | 117 | 105-163 |
| IIV V_p_/F (%) | 25.5 | 14.1-31.6 | - | - |
| IIV ARU (%) | 53.3 | 24.4-71.0 | 47.4 | 35.0-54.4 |
| Covariance IIV CL/F-V_c_/F (%) | 91.7 | 78.1-92.0 | 90.9 | 81.8-90.2 |
| *Residual error* |  |  |  |  |
| Proportional Plasma (%) | 37.5 | 32.5-42.1 | 21.0 | 17.3-22.0 |
| Proportional Urine (%) | 58.4 | 53.9-62.3 | 58.7 | 52.3-64.8 |
| Additive Plasma (mg/L) | - | - | 0.004 | 0.003-0.005 |
| Additive Urine (mg/L) | - | - | 0.006 | 0.003-0.007 |

^1^ 90% percentile CI from nonparametric bootstrap (1000 samples)

CL/F: apparent oral clearance; V_c_/F, V_p_/F: apparent central and peripheral volume of distribution; Q/F: intercompartmental clearance; k_a_: absorption rate constant;

ARU: urine accumulation ratio (proportionality constant); IIV: interindividual variability
